# Modulating the cholinergic system—Novel targets for deep brain stimulation in Parkinson's disease

**DOI:** 10.1111/jnc.16264

**Published:** 2024-11-18

**Authors:** V. Witzig, R. Pjontek, S. K. H. Tan, J. B. Schulz, F. Holtbernd

**Affiliations:** ^1^ Department of Neurology RWTH Aachen University Aachen Germany; ^2^ Department of Neurosurgery RWTH Aachen University Aachen Germany; ^3^ Department of Stereotactic and Functional Neurosurgery University Hospital Cologne Cologne Germany; ^4^ Department of Neurosurgery Antwerp University Hospital Edegem Belgium; ^5^ Translational Neurosciences, Faculty of Medicine and Health Sciences University of Antwerp Antwerp Belgium; ^6^ JARA‐BRAIN Institute Molecular Neuroscience and Neuroimaging Jülich Research Center GmbH and RWTH Aachen University Aachen Germany; ^7^ Jülich Research Center, Institutes of Neuroscience and Medicine (INM‐4, INM‐11) Jülich Germany

**Keywords:** acetylcholine, deep brain stimulation, neuromodulation, nucleus basalis of Meynert, Parkinson's disease, pedunculopontine nucleus

## Abstract

Parkinson's disease (PD) is the second‐fastest growing neurodegenerative disease in the world. The major clinical symptoms rigor, tremor, and bradykinesia derive from the degeneration of the nigrostriatal pathway. However, PD is a multi‐system disease, and neurodegeneration extends beyond the degradation of the dopaminergic pathway. Symptoms such as postural instability, freezing of gait, falls, and cognitive decline are predominantly caused by alterations of transmitter systems outside the classical dopaminergic axis. While levodopa and deep brain stimulation (DBS) of the subthalamic nucleus or globus pallidus internus effectively address PD primary motor symptoms, they often fall short in mitigating axial symptoms and cognitive impairment. Along these lines, the cholinergic system is increasingly recognized to play a crucial role in governing locomotion, postural stability, and cognitive function. Thus, there is a growing interest in bolstering the cholinergic tone by DBS of cholinergic targets such as the pedunculopontine nucleus (PPN) and nucleus basalis of Meynert (NBM), aiming to alleviate these debilitating symptoms resistant to traditional treatment strategies targeting the dopaminergic network. This review offers a comprehensive overview of the role of cholinergic dysfunction in PD. We discuss the impact of PPN and NBM DBS on the management of symptoms not readily accessible to established DBS targets and pharmacotherapy in PD and seek to provide guidance on patient selection, surgical approach, and stimulation paradigms.

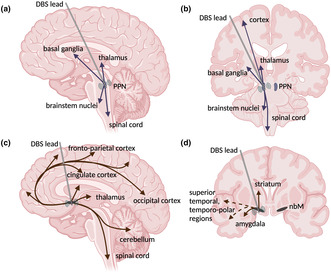

## Graphical abstract

Parkinson's disease (PD) is not only associated with motor deficits due to dopaminergic dysfunction but also with cognitive and gait impairment driven by cholinergic system degeneration. This review highlights deep brain stimulation (DBS) of cholinergic structures like the pedunculopontine nucleus and nucleus basalis of Meynert as novel targets and provides guidance on application. Modulating the cholinergic system via DBS could expand treatment options for PD patients, offering relief for debilitating symptoms such as freezing of gait and cognitive impairment, which dopaminergic therapies cannot effectively manage.

**Abbreviations**
ACh: acetylcholine
AChEIs: acetylcholine esterase inhibitors
AD: Alzheimer's disease
A‐syn: alpha‐synuclein
CINs: cholinergic interneurons
DBS: deep brain stimulation
DLB: dementia with Lewy body
DREADDs: designer receptors exclusively activated by designer drugs
FOG: freezing of gait
GABA: gamma aminobutyric acid
GPi: globus pallidus internus
HFS: high‐frequency stimulation
LD: levodopa
LFS: low‐frequency stimulation
MRI: magnetic resonance imaging
MSN: medium spiny neurons
NBM: nucleus basalis of Meynert
PD: Parkinson's disease
PDD: Parkinson's disease dementia
PET: positron emission tomography
PPN: pedunculopontine nucleus
SNc: substantia nigra pars compacta
SNr: substantia nigra pars reticulata
STN: subthalamic nucleus
UPDRS: Unified Parkinson's Disease Rating Scale

## 1 INTRODUCTION

Parkinson's disease (PD) is a progressive neurological disease characterized by motor symptoms, primarily resulting from the alpha‐synuclein (α‐syn) induced loss of dopaminergic neurons in the substantia nigra pars compacta (SNc) and their striatal terminals (Braak et al., 2004). In 2016, nearly 6.1 million people worldwide were affected by PD and the global burden of the disease has nearly doubled over the last years (Dorsey et al., 2018).

Virtually all PD patients (98.6%) suffer from at least one non‐motor symptom, and approximately 75% of patients who survive more than 10 years will develop dementia (Aarsland & Kurz, 2010; Barone et al., 2009). Severe cognitive impairment usually emerges at later disease stages in PD (Aarsland et al., 2021). In contrast, dementia with Lewy bodies (DLB) should be suspected in patients presenting with cognitive impairment manifesting before or concomitant with motor onset. PD dementia (PDD) and DLB share many clinical, neurochemical, and morphological features. Both are characterized by pathological α‐syn aggregates, neuronal loss, and neurite Lewy body inclusions in midbrain neurons (Bohnen & Albin, 2011). In clinical practice, it can be challenging to distinguish PDD from DLB. According to the current Movement Disorder Society diagnostic guidelines, the distinction between both entities is still based solely on the time elapsed between the emergence of cognitive and motor symptoms, respectively (i.e., in DLB, cognitive symptoms occur before or within 12 months after the onset of parkinsonism) (McKeith et al., 2017). Even though distinct alterations of neurochemistry and histopathology have been reported for DLB and PDD (Bohnen & Albin, 2011; Jellinger, 2018), there is substantial pathophysiological overlap, and most likely PDD and DLB present different ends of an α‐syn induced neurodegenerative process (Jellinger, 2018).

**C**ognitive function, but also gait, and posture rely on the integrity of the cholinergic system. Importantly, there is a close interplay between dopaminergic and cholinergic transmission in the basal ganglia. For example, reduced dopaminergic input from the SNc in PD drives cholinergic hyperactivity in the striatum, which explains why anticholinergic drugs improve motor symptoms, and tremor in particular (Calabresi et al., 2006; Clarke, 2004). In addition to striatal dysbalance, α‐syn related neurodegeneration of the pedunculopontine nucleus (PPN) and the nucleus basalis of Meynert (NBM) cause a decrease of the cholinergic tone (Bohnen et al., 2009). Alongside the striatum, the PPN and the NBM are the two main cholinergic resources of the highly complex and largest neurotransmitter system in the central nervous system (Bohnen & Albin, 2011; He et al., 2023). In advanced stages of PD, cognitive and gait dysfunction are frequent and often burdensome (Lieberman et al., 2019; Perez‐Lloret et al., 2014; Zhang et al., 2021). Gait is particularly impaired during dual task exercises, suggesting that gait dysfunction has a relevant cognitive component (Bohnen et al., 2013; O'Shea et al., 2002; Yogev et al., 2005). Decreased nigro‐striatal dopaminergic integrity and α‐syn induced pathology of subcortical and cortical cholinergic resources in concert are important drivers of cholinergic dysfunction underlying these often debilitating symptoms in PD (Calabresi et al., 2006).

Drug and surgical treatments of PD primarily focus on improving motor symptoms (Nemade et al., 2021). Although dopamine replacement therapy is successful for most motor symptoms (Nemade et al., 2021), it has limited effects on axial motor symptoms, such as postural instability, FOG, and falls (Smulders et al., 2016). If axial symptoms are unresponsive to dopaminergic treatment, pharmacological options are often futile. However, there is evidence that administration of AChEIs may improve falls and gait to some extent (Henderson et al., 2016). Furthermore, the evidence for non‐pharmacological treatment with effects on gait has grown in recent years (Delgado‐Alvarado et al., 2020). FOG can be ameliorated by passive treatment options, such as non‐invasive stimulation techniques (i.e., repetitive transcranial magnetic stimulation; Kim et al., 2015), or active treatment options like physical training (Canning et al., 2015) and cognitive programs (Fietzek et al., 2014). These active and passive treatment options have been shown to induce long‐lasting effects on gait. The use of anticholinergics in PD to ameliorate motor symptoms is often limited by severe cognitive deterioration (Perez‐Lloret et al., 2016). In fact, cognitive impairment in PD can effectively be treated by strengthening the cholinergic system using acetylcholine esterase inhibitors (AChEIs) such as rivastigmine (Emre et al., 2004). In contrast, the application of dopaminergic medication has shown disappointing results in treating cognitive symptoms (Calabresi et al., 2006; Kulisevsky et al., 2000). Non‐pharmacological treatment options and their effects on cognition in PD patients have been reviewed lately (Pupíková & Rektorová, 2020). While cognitive training with the aim to improve performance in attention and working memory showed the highest evidence for having cognitive effects (Fellman et al., 2020; París et al., 2011; Petrelli et al., 2014), physical training or non‐invasive brain stimulation techniques cannot be recommended based on the current literature (Altmann et al., 2016; Gobbi et al., 2013; Hashimoto et al., 2015; Pupíková & Rektorová, 2020; Shinichi Amano & Chris J Hass, 2013; Silveira et al., 2018).

Beyond these treatment options, deep brain stimulation (DBS) has become an established neurosurgical treatment for PD, which leads to sustained improvement of motor function and quality of life (Lozano et al., 2019). Although the exact mechanisms of DBS remain elusive, several theories have been proposed. Normalizing the firing rate of pathological activity patterns (e.g., beta‐band oscillations) and thereby regulating an overactive basal ganglia system is currently regarded as the most promising hypothesis (Agnesi et al., 2013; Alosaimi et al., 2022; Ashkan et al., 2017). However, these theories neglect global stimulation effects in remote areas from the stimulation site. In this context, the concept of a modulatory stimulation effect of non‐dopaminergic neurotransmitter systems has gained increasing interest (Alosaimi et al., 2022; Lozano et al., 2019). Indeed, DBS targeting the PPN and NBM has emerged as a promising strategy to enhance the cholinergic tone and consequently ameliorate gait disturbances and cognitive deficits in PD, which are otherwise often difficult to treat (Bohnen, Yarnall, et al., 2022). PPN and NBM DBS have been explored in animal studies, small case series, and few randomized controlled trials, providing promising results. However, studies directly comparing different stimulation paradigms are scarce, and clear recommendations for clinical application are lacking. Thus, patient and target selection and the identification of the most efficient stimulation paradigm for optimal clinical outcome remain challenging. Here, we provide a concise overview of proposed concepts of cholinergic modulation by NBM and PPN DBS in PD. We discuss the utility of targeting the cholinergic system to improve axial and cognitive symptoms and seek to provide guidance for patient selection, surgical approach, and stimulation paradigms.

## 2 ANATOMY OF THE CHOLINERGIC SYSTEM AND CHANGES IN PD

The PPN, NBM, and striatum are the key nodes of the cholinergic neurotransmitter system (Bohnen & Albin, 2011).

### 2.1 The PPN

The PPN lacks clear anatomical definition and predominantly consists of cholinergic (25%–30%) and glutamatergic (40%–45%) neurons (Tubert et al., 2019). Historically, it has been categorized into the caudal pars compacta and the rostral pars dissipata based on its cytoarchitecture and neurochemical markers (Geula et al., 1993; Lin et al., 2023; Mesulam et al., 1983; Pienaar et al., 2017). Most cholinergic neurons are found in the caudal part of the PPN (Pahapill & Lozano, 2000). The dorsal part of the PPN initiates movement, while the ventral part coordinates the stopping (Lin et al., 2023; Sherman et al., 2015). The PPN is crucial to governing gait (Gut & Mena‐Segovia, 2019; Ricciardi et al., 2019). However, it also belongs to the reticular activating system and is involved in controlling the sleep–wake cycle. For example, PPN dysfunction has been associated with daytime sleepiness and rapid eye movement sleep behavior disorder pathophysiology (Boeve et al., 2004; Chambers et al., 2020). Its connection with cortical areas like the somatosensory and presupplementary motor areas underline its role in cognitive and motivational processes. Degeneration of the PPN leads to impairment in attention, motivation and compulsive behaviors (French & Muthusamy, 2018). It has been suggested that the PPN plays a modulatory role in motor control, mainly by influencing both cognitive and behavioral functions by shaping reward signaling and adaptive behavior. This modulatory role is crucial for sensorimotor integration. Alterations of this delicate system play an important role in the development of gait impairment in PD (Gut & Mena‐Segovia, 2019).

**FIGURE 1:**
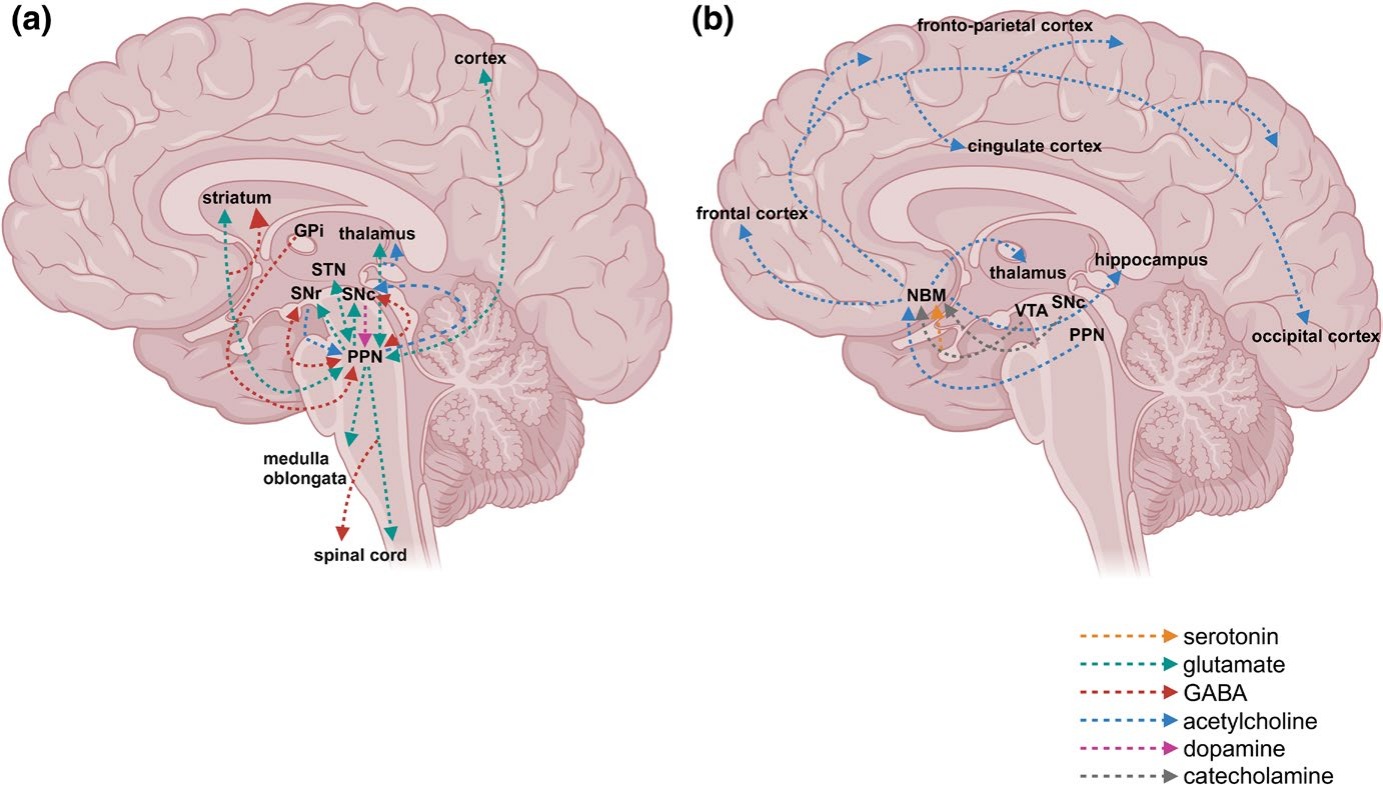
Major efferent connections of pedunculopontine nucleus (PPN) and nucleus basalis of Meynert (NBM) in deep brain stimulation (DBS). The PPN exhibits extensive glutamatergic and cholinergic projections to the cortex, thalamus, basal ganglia, limbic structures, several brainstem nuclei, and the spinal cord (a, b). The largest region of the NBM (Ch4‐subregion) innervates the limbic region, including the cingulate cortex, the frontoparietal cortex, and the amygdala (c, d). Furthermore, it establishes connections with the superior temporal and temporal Polar Regions (c, d).

On an electrophysiological level, based on spontaneous activity (3–16 Hz) with regular spiking, cholinergic neurons in the PPN are characterized as type II neurons, showing a high density of outward rectifier Kv4‐potassium channels (Takakusaki & Kitai, 1997; Tubert et al., 2019). The PPN is integral to a circuit governing movement. Both the afferent and efferent connections are complex (displayed in Figure 1a,b) and have been the subject of several reviews (Lin et al., 2023; Tubert et al., 2019). The PPN receives substantial input from the basal ganglia, notably gamma aminobutyric acid (GABA) projections from the substantia nigra pars reticulata (SNr) and the globus pallidus internus (GPi). Additionally, it receives dopaminergic input from the SNc, and glutamatergic projections from the subthalamic nucleus (STN). Furthermore, it receives excitatory input from the motor cortex, and deep cerebellar and midbrain nuclei (Lin et al., 2023). The PPN has glutamatergic and cholinergic efferent connections to various regions, including the cortex, thalamus, basal ganglia, limbic structures, several brainstem nuclei, and the spinal cord (Tubert et al., 2019). One of the best studied efferent connections is the direct excitatory glutamatergic projection of the PPN to dopaminergic neurons in the SNc, which was studied in electrophysiological experiments using organotypic rodent brain slices (Futami et al., 1995) and other ex vivo studies (Di Loreto et al., 1992; Scarnati et al., 1986, 1987; Tubert et al., 2019).

### 2.2 The NBM

The NBM is located in the basal forebrain above and parallel to the optic nerve, with its medial border being the lateral ventricle (Liu et al., 2015). Four cholinergic “cluster” cell groups (Ch1–Ch4) without distinct boundaries have been identified from studies in non‐human primates using immunohistochemistry and histochemistry (Mesulam et al., 1983, 1984). Ch1 (medial septal nucleus) and Ch2 (vertical limb of the diagonal band nucleus) both project to the hippocampal complex (Liu et al., 2015). Ch3 (horizontal limb of the diagonal band nucleus) projects to the olfactory bulb (Liu et al., 2015). The largest is the Ch4 subgroup, which exhibits cellular variations and can be subdivided into further subgroups projecting to different cortical regions and the amygdala. Specifically, the anterior Ch4 subregion innervates the limbic region, with the anteromedial region projecting to the cingulate cortex, while the anterolateral Ch4 subregion projects to the frontoparietal cortex and the amygdala (Figure 1c,d). The posterior Ch4 subregion establishes connections with the superior temporal and temporal polar regions (Liu et al., 2015) (Figure 1c,d). Notably, these cortical NBM connections are reciprocal. The NBM lacks anatomically defined borders of the subregions. Therefore, in the human brain, the classification of the Ch4 subregion has been simplified to an anterior, intermediate, and a posterior subregion (Liu et al., 2015). The NBM plays a crucial role in memory, arousal, attention, and perception (Goard & Dan, 2009). There is mounting evidence from both histopathological and imaging studies linking NBM atrophy to cognitive impairment in PD in cross‐sectional and longitudinal study designs (Gang et al., 2020; Palmer et al., 1987; Schulz et al., 2018; Whitehouse et al., 1983). Moreover, recent studies have suggested that structural alterations of the NBM are predictive of cognitive decline in prodromal PD (Zhang et al., 2024). That said, cholinergic cell death in the NBM is likely not the sole driver of cognitive decline in PD. For example, meta‐analyses of imaging data clearly demonstrated widespread cortical atrophy in demented PD patients, whereby the insular cortex and hippocampi emerged as sites of most severe volume loss (Mihaescu et al., 2019). It remains unclear if and to what extent cholinergic dysfunction is responsible for these findings. Nevertheless, there is little doubt that NBM dysfunction is closely related to cognitive impairment in PD (Bohnen & Albin, 2011), but alterations of other neurotransmitters such as serotonin may also contribute to cognitive impairment (Prado et al., 2017). Over three decades ago, stimulation of the NBM has demonstrated an increase in cortical cholinergic release (Kurosawa et al., 1989a). Consequently, there is a renewed interest in NBM DBS for addressing cognitive dysfunction in Alzheimer's disease (AD), DLB, and PDD (Bohnen, Yarnall, et al., 2022; Gratwicke et al., 2013, 2020; Nazmuddin et al., 2021).

### 2.3 The striatum

While only 1%–2% of neurons in the striatum are cholinergic, these cholinergic interneurons (CINs) provide the highest density of cholinergic markers in the brain (Bohnen & Albin, 2011). CINs are large (20–50 μm) aspiny neurons. The balance of dopaminergic and cholinergic transmitter systems is crucial in maintaining striatal microcircuitry for the control of movement and cognition in the physiological state (Ztaou & Amalric, 2019). CINs receive dopaminergic input from the SNc and the PPN and synapse within the striatum on GABAergic medium spiny neurons (MSNs), the latter of which form the largest neuronal population in the striatum (Calabresi et al., 2006; Izzo & Bolam, 1988). Inhibitory and excitatory signaling among CINs and MSNs is mediated by dopaminergic (D1 and D2), muscarinergic, and nicotinergic receptors (Calabresi et al., 2006; Sealfon & Olanow, 2000). MSNs in the direct pathway are activated by dopamine via dopaminergic D1 receptors and inhibited by acetylcholine (ACh) via muscarinergic M4 receptors, while indirect MSNs are inhibited by dopamine via D2 receptors and activated by ACh via muscarinergic M1 receptors. All CINs express D2‐receptors, while only a small portion expresses D1 receptors (Gonzales & Smith, 2015; Lim et al., 2014). Both receptors are involved in the functional circuitry of working memory (Castner et al., 2000; Wang et al., 2004). Dopamine exerts an inhibitory effect on CINs via D2‐receptors which leads to a decrease of striatal ACh release (Abercrombie & DeBoer, 1997; Consolo et al., 1993; Lehmann et al., 1983; Pisani et al., 2000; Stoof et al., 1992; Yan et al., 1997), while dopamine increases cholinergic transmitter concentrations via activation of D1‐receptors (Abercrombie & DeBoer, 1997; Acquas & Di Chiara, 1999; Damsma et al., 1991; Di Chiara et al., 1994; Steinberg et al., 1998). Together, dopaminergic and cholinergic receptors build synaptic strength and plasticity (Calabresi et al., 2006). Long term potentiation and depression are two forms of synaptic plasticity and are regarded as a model for storage and retrieval of neuronal information (Calabresi et al., 2006). The complex structural and functional striatal‐cortical interaction is crucially involved in executive function, including planning movement and goal directed behavior (Calabresi et al., 2006).

### 2.4 A‐syn pathology in PD and the cholinergic system

While pathological α‐syn aggregation has been identified as a hallmark of PD pathology, these aggregates are not unique to this disease. Abnormal formation of α‐syn fibrils can also be found in atypical parkinsonian syndromes (e.g., multisystem atrophy or pure autonomic failure), presenting with unique clinical phenotypes (Calabresi et al., 2023). Furthermore, rapid eye movement sleep behavior disorder has been recognized as a prodromal state of alpha synucleinopathies such as PD and multisystem atrophy. According to the Braak stages of PD, α‐syn aggregates form in the glossopharyngeal and vagal nerve, and the anterior olfactory bulb (stage 1), proceed into the dorsal raphe nuclei and magnocellular portions of the reticular formation (stage 2), until pathology reaches the midbrain and forebrain including the pontine tegmentum (stage 3). Finally, α‐syn aggregates reach the remaining subcortical areas and the cortex (stages 4–6) (Braak et al., 2003). This widespread retrograde propagation of α‐syn throughout the entire brain implicates that neuropathological degeneration in PD extends beyond the degeneration of the dopaminergic nigrostriatal pathway and affects various other neurotransmitter systems. Recent studies suggest that the spread of α‐syn does not occur uniformly, and that regional differences in α‐syn pathology derive from selective neuronal vulnerability to α‐syn aggregation. Along these lines, the extent of dendritic arborization and neuronal electrophysiological properties lead to an increased vulnerability to age, environmental toxins, and gene mutations (Surmeier et al., 2017). For instance, dopaminergic neurons in the SNc are characterized by long, unmyelinated and highly branched axons with many transmitter release sites, which is correlated with oxidative stress and a higher uptake of α‐syn aggregates (Braak et al., 2004; Pacelli et al., 2015; Surmeier et al., 2017). However, striatal CINs also express long and highly branched axons but do not degenerate to such an extent in the parkinsonian brain (Surmeier et al., 2017). Therefore, besides morphology, other mechanisms leading to a higher vulnerability must exist. Dopaminergic neurons in SNc are autonomous pacemakers and fire in a slow, tonic pattern creating high cytosolic concentrations of calcium (Guzman et al., 2009; Puopolo et al., 2007; Surmeier et al., 2012). Especially the slow calcium oscillations (Puopolo et al., 2007; Putzier et al., 2009) and high cytosolic and mitochondrial calcium concentrations (Guzman et al., 2010; Hayashi et al., 2009; Sanchez‐Padilla et al., 2014) are considered as tipping points differentiating between physiological condition and the beginning of Lewy body disease (Surmeier et al., 2017). It is unclear whether similar changes are present in cholinergic neurons, and thus may contribute to differences in cellular vulnerability. That said, a recently published study exploring α‐syn vulnerability of striatal neuronal populations in mice found a comparable magnitude of α‐syn aggregation and related cell death in SNc dopaminergic neurons compared with PPN cholinergic neurons after local α‐syn injection into the SNc and PPN, respectively (Geibl et al., 2024).

#### 2.4.1 Degeneration of the PPN in PD

The presence of Lewy body formation is accompanied by neurodegeneration in the PPN. Because of neurodegeneration and excessive descending inhibition from the GPi and SNr, the PPN is less active (Thevathasan & Moro, 2019). Specifically, neuronal recordings from PPN electrodes have revealed a decrease in α‐band activity in the PD brain. α‐band activity is positively associated with gait performance and is detectable especially in the caudal part of the PPN. Decreased α‐band activity in the PPN has been associated with FOG in PD patients (Thevathasan & Moro, 2019).

In PD, 40%–70% of cholinergic neurons in the lateral PPN are lost because of neurodegeneration, leading to deficits in gait and posture (Bohnen et al., 2009, 2013, 2019; Chambers et al., 2021; Gai et al., 1991; Hirsch et al., 1987; Jellinger, 1988; Rinne et al., 2008; Zweig et al., 1989). Until recently, studies of changes in the cholinergic system in PD have largely been based on post‐mortem autopsy studies. Today, modern molecular imaging methods including single photon emission computed tomography, positron emission tomography (PET), and magnetic resonance imaging (MRI) allow for non‐invasive correlations of cholinergic degeneration and structural changes with clinical symptoms (Albin et al., 2022). For example, the degree of cholinergic neuronal loss in the PPN is more prominent in PD individuals who experience falls compared with those who do not (Karachi et al., 2010; Nardone et al., 2017). Moreover, the degeneration of cholinergic neurons has been associated with cognitive deficits, particularly with attentional and executive dysfunction, which has led to the hypothesis that impaired gait‐balance in PD may be predominantly caused by deficits of the attentional cognitive domain (Albin et al., 2022).

#### 2.4.2 Degeneration of the NBM in PD

In PD, up to 80% of cholinergic cells in the NBM are depleted (Liu et al., 2015), particularly the large neuronal population within the Ch4 subregion (Hall et al., 2014). The extent of cholinergic neuron loss in the NBM in PD is comparable to AD cases, and pronounced in PDD patients (Hall et al., 2014; Liu et al., 2015). The cholinergic loss and concomitant atrophy may serve as predictive markers for the severity of cognitive deficits in PD as demonstrated by a significant correlation with cognitive impairment in several studies (Choi et al., 2012; Gratwicke et al., 2015; Gratwicke & Foltynie, 2018; Shimada et al., 2009; Whitehouse et al., 1983). Along these lines, NBM atrophy, in particular of the Ch4 subregion, identified via MRI volumetric studies, correlated with lower Montreal Cognitive Assessment (Gill et al., 2008; Zadikoff et al., 2008) test results (Gratwicke & Foltynie, 2018). Of note, the extent of NBM atrophy in PD is comparable to that observed in AD (Candy et al., 1983). A recent study using [^18^F]‐fluoroethoxybenzovesamicol vesicular ACh transporter PET and post‐mortem volumetric MRI found that the ACh concentration in the basal forebrain of 101 non‐demented PD patients correlated with NBM volume (Ray et al., 2023). Interestingly, ACh binding in the basal forebrain differs between cognitively impaired and cognitively intact PD patients. In one study, 57 PD patients were divided into a subgroup of patients with mild cognitive impairment and another subgroup of patients without cognitive dysfunction (Van Der Zee, Kanel, Gerritsen, et al., 2022). Both groups presented lower cortical ACh binding than the control group (Van Der Zee, Kanel, Gerritsen, et al., 2022). However, there was a higher than normal ACh binding in de novo PD patients in cortical and subcortical subregions (Van Der Zee, Kanel, Gerritsen, et al., 2022). These findings have led to the hypothesis that an initial up‐regulation of ACh in the early stages of the disease may compensate for nigrostriatal dopaminergic degeneration to maintain cognitive function (Bohnen et al., 2012; Sarter et al., 2014; Van Der Zee, Kanel, Gerritsen, et al., 2022; Van Der Zee, Kanel, Müller, et al., 2022). As cholinergic degeneration emerges, this mechanism ultimately fails, and cognitive decline is inevitable (Bohnen, Roytman, et al., 2022). Neurodegeneration in the NBM is likely a result of α‐syn pathology (Del Tredici & Braak, 2016; Selden, 1998). NBM dysfunction is believed to start prior to NBM atrophy, probably because of α‐syn‐associated inflammatory processes (Rocha et al., 2018). Inflammation can be imaged by assessing the extracellular free water fraction on diffusion weighted MRI (Febo et al., 2020; Pasternak et al., 2009). Of note, increases of the free water fraction correlated with measures of executive dysfunction (an early symptom of cognitive decline in PD), while NBM volume correlated with memory impairment (a late symptom of cognitive decline in PD). These findings suggest a stepwise progression of cholinergic dysfunction and associated cognitive decline in PD that can be assessed by different imaging modalities (Crowley et al., 2023). Another clinical trial using C‐methyl‐4‐piperidinyl propionate, which reflects cortical acetylcholinesterase activity, reported reduced posterior basal forebrain volume only in those PD patients who exhibited reduced cortical acetylcholinesterase activity. Similarly, patients with mild cognitive impairment showed lower cortical acetylcholinesterase activity (Schumacher et al., 2023). These results further prove the tight relationship between NBM volume loss, cholinergic deficiency, and cognitive decline in PD.

#### 2.4.3 Degeneration of the striatum in PD

Striatal interneurons seem to be partially spared by neurodegeneration in PD (Calabresi et al., 2006). This has been attributed to a relative low vulnerability to α‐syn pathology and increased axonal sprouting of CINs as a result of dopaminergic deafferentation from the SNc, leading to an imbalance between the cholinergic and dopaminergic transmitter systems (Calabresi et al., 2006; Spehlmann & Stahl, 1976). Dopaminergic cell loss in PD reduces dopamine levels in the striatum and induces a shift toward the indirect pathway. As a result, CINs become more excitable, excessively release ACh, and induce synaptic reorganization (Ztaou & Amalric, 2019). The reduction of striatal dopamine partly explains the dysexecutive syndrome typical of PD, caused by an imbalance of mechanisms implied in synaptic plasticity, including long‐term potentiation, depression, and synaptic depotentiation (Calabresi et al., 2006). Accordingly, PD patients experience difficulties in tasks that require cognitive flexibility, such as adapting to new situations or making new strategies. Dopamine replacement therapy has been shown to improve some aspects of executive dysfunction in early PD (Cooper et al., 1992). However, dopaminergic treatment falls short in mitigating cognitive dysfunction on a more global level (Brusa et al., 2005; Kulisevsky et al., 2000).

## 3 MODULATING THE CHOLINERGIC SYSTEM BY DBS

DBS of the STN and GPi improves PD‐related motor symptoms such as rigor, tremor, and bradykinesia (Bohnen, Yarnall, et al., 2022; Bove et al., 2021; Lozano et al., 2019). However, effects on postural instability, falls, and FOG are limited (Brozova et al., 2021; Castrioto, 2011; Lozano et al., 2019; St. George et al., 2010). There are several reviews which have highlighted the association between an increased cholinergic tone and the improvement of gait, balance, and falls. For instance, Morris et al. have recently reviewed pharmacological, imaging, and electrophysiological data to investigate the role of ACh in axial symptoms. The strongest associations were described for gait speed and falls with increased or decreased cholinergic levels, respectively (Morris et al., 2019).

Both NBM and PPN DBS are generally well tolerated, and few adverse events have been reported. Side effects of both targets include the known stimulation‐associated voltage side effects which can include oscillopsia, paresthesia, burning sensations, myoclonus, sleep induction, and incontinence (Hamani et al., 2011). In most cases, these side effects can be resolved by the adjustment of stimulation parameters. Data on severe complications such as intracranial hemorrhage are insufficient to allow for comparisons with traditional DBS targets (Welter et al., 2015). That said, the majority of reports available do not suggest a high rate of severe adverse events independent of the target (PPN or NBM) or surgical approach (single vs. multiple targets) (Ferraye et al., 2010; Gratwicke et al., 2018, 2020; Maltête et al., 2021; Mazzone et al., 2005; Picton et al., 2024; Stefani et al., 2007).

DBS of the striatum, especially the caudate nucleus, has been discussed controversially, as previous STN DBS trials have attributed poor cognitive outcomes to accidental lead placement through the caudate nucleus (Isler et al., 2016; Morishita et al., 2014; Witt et al., 2012, 2013). However, these negative effects have been refuted by more recent studies (Bot et al., 2018; Tesio et al., 2019). Stimulation of the ventral striatum (nucleus accumbens) has shown some merit in a few cases of dystonia and Tourette syndrome (Johnson et al., 2019), and diseases caused by disrupted reward circuits, such as obsessive compulsive disorder (Mantione et al., 2010), eating disorder (Mantione et al., 2010), and obesity (Lee et al., 2019; Mantione et al., 2010). Few data, stemming from animal experiments, are available on the effect of striatal DBS. Recently, D1‐type receptors of striatal MSNs were targeted by cell specific optogenetic stimulation in dopamine‐depleted mice (Kim et al., 2022). Stimulation influenced brain rhythms, especially in the dorsal striatum and when applying low frequency stimulation (LFS: 4 Hz), leading to significant improvement of head movement in dopamine depleted mice. The effects of LFS and HFS on cortico‐striatal synapses regarding LD‐induced dyskinesia were previously investigated in rats. LD‐induced dyskinesia is believed to rely on cortico‐striatal synapses. Rats were rendered hemi‐parkinsonian and consecutively chronically treated with LD. Whereas some rats showed motor improvement without dyskinesia, others developed debilitating dyskinesia in response to LD treatment. HFS induced long‐term potentiation in both groups, while LFS led to depotentiation in the dyskinetic group only (Calabresi et al., 2006; Picconi et al., 2003). HFS (200 Hz) of the anterior caudate nucleus in rhesus monkeys has been shown to enhance learning, especially in the acquisition of specific visuomotor associations (Williams & Eskandar, 2006), while negative effects on cognition were observed following HFS in the dorsal striatum of rats (Schumacher et al., 2011). Despite these encouraging reports, the striatum is not an established DBS target. Furthermore, to our knowledge, there are no trials available exploring the effect of striatal DBS in PD, including the cholinergic system. This may derive from several reasons. Foremost, the striatum is a large, complex and functionally diverse structure with only 1%–2% of cholinergic neurons. It plays a critical role in multiple neural circuits, including those involved in motor control, reward processing, habit formation, and cognitive flexibility (French & Muthusamy, 2018). Targeting a specific part of the striatum without affecting neighboring areas or circuits is difficult. Small variations in electrode placement may lead to different outcomes or unintended side effects.

### 3.1 PPN DBS

The PPN has been proposed as a target to alleviate axial symptoms and has been studied extensively in pre‐clinical and clinical studies with promising results (Jenkinson et al., 2005; Mazzone et al., 2005; Plaha & Gill, 2005; Stefani et al., 2007).

#### 3.1.1 Animal studies

The PPN receives inhibitory GABAergic afferences from the GPi and the substantia SNr (Lin et al., 2023). Thus, manipulating the GABAergic input is considered a strategy to disinhibit the PPN and, consequently, alleviate akinesia. Notably, microinjections of bicuculline (a GABA receptor‐A antagonistic substance) in a non‐human primate 1‐methyl‐4‐phenyl‐1,2,3,6‐tetrahydropyridine PD model, improved motor function comparable to LD treatment (Nandi et al., 2002). This pioneer work highlighted the PPN as a potential stimulation target for medication refractory gait and posture disability in advanced PD (Nandi et al., 2002). Subsequent experiments employed PPN DBS in macaques and other non‐human primates. Akinesia was induced by unilateral PPN stimulation at high frequencies, ranging from 45 to 100 Hz. Facial expression, limb and body motion, and behavior of monkeys were assessed using video recordings (Nandi et al., 2002). Similarly, Jenkinson et al. demonstrated that unilateral HFS (100 Hz) reduced motor activity in monkeys, which was assessed with a partially blinded motor score after video recording of motor behavior. In contrast, unilateral LFS (5 Hz) had adverse effects, prompting movement and reversing akinesia (Jenkinson et al., 2004). In rodents, most PPN connections exist bilaterally, but have a dominant side. Pre‐clinical findings have suggested that, even though unilateral stimulation has an effect on the contralateral side, bilateral stimulation might be more effective (Hamani et al., 2016). These studies have furthered the idea of a more effective low‐frequency, bilateral stimulation. This concept has subsequently been translated into human studies.

Given the co‐existence of glutamatergic, GABAergic, and cholinergic neurons in the PPN, the question arises whether the effects of PPN DBS are indeed mediated by modulation of cholinergic neurotransmission. Along these lines, Wen et al. performed microdialysis experiments to assess cholinergic transmitter levels in brain tissue of a common PD animal model. Injections of 6‐hydroxydopamine into the medial‐forebrain bundle of rats reduced concentrations of ACh in the ventrolateral thalamic nucleus. The observed changes of transmitter levels correlated with a Parkinson phenotype characterized by reduced stride length, reduced maximum area of paw contact on the floor, and reduced base of support (average width between either the front or the hint paw) (Wen et al., 2015), suggesting a strong association between gait impairment and low cholinergic transmitter levels. Importantly, unilateral LFS (25 Hz) of the PPN increased ACh levels, which was accompanied by an improvement of gait observed on CatWalk gait analysis (Wen et al., 2015). We evaluated the impact of chronic STN DBS on the cholinergic system in a 1‐methyl‐4‐phenyl‐1,2,3,6‐tetrahydropyridine mouse model. In line with the previous study, 1‐methyl‐4‐phenyl‐1,2,3,6‐tetrahydropyridine reduced choline acetyltransferase‐positive neurons in the PPN compared to saline treatment. Mice exhibited a Parkinson phenotype as assessed by walking tests. Gait impairment was reversed by STN DBS. However, STN DBS did not alter the number of activated choline acetyltransferase expressing neurons in the depleted PPN. This suggests that STN‐stimulation likely did not improve gait via the modulation of the remaining cholinergic PPN neurons (Witzig et al., 2023). Along these lines, a structural connectivity study showed that gait improvements in STN DBS treated PD patients were linked to stimulation of fiber tracts connecting the STN and motor cortex (Gradinaru et al., 2009). Similarly, optogenetic stimulation of the STN has attributed the motor benefits of STN stimulation to the modulation of upstream connections between the STN and frontal cortices (Strelow et al., 2021). Thus, the positive effects on gait in our study likely were because of stimulation of fiber tracts outside the cholinergic system. That said, it is important to point out that STN DBS often fails to alleviate gait dysfunction and even can induce gait impairment in PD (Brozova et al., 2021; Castrioto, 2011; Lozano et al., 2019; St. George et al., 2010).

While evidence for a direct modulation of cholinergic transmission via PPN DBS is limited, there is strong evidence for a close relationship of PPN cholinergic integrity and gait disturbances in PD. In this vein, a vesicular ACh transporter knock‐out mouse model was used to study cholinergic neurotransmission in the midbrain (Janickova et al., 2017). Modified mice lacked the vesicular ACh transporter in the pedunculopontine and laterodorsal tegmental nuclei cholinergic neurons and showed impaired motor learning and coordination deficits in a standardized gait balance test (rotarod test), moved slower, and presented smaller steps on the catwalk test. These symptoms worsened with aging but reached a ceiling effect, highlighting the dominant role of PPN cholinergic neurons in gait control (Janickova et al., 2017). Another line of research, emphasizing the pivotal role of PPN cholinergic neurons in the mediation of axial symptoms employed artificially engineered protein receptors (designer receptors) selectively targeted by certain ligands (so‐called designer drugs (DREADDs)) to detect specific cell‐types, which are activated by PPN DBS. Choline acetyltransferase transgenic mice rendered parkinsonian by intra‐nigral, monohemispheric stereotaxic administration of the ubiquitin‐proteasomal system inhibitor, lactacystin, received DREADDs to transiently activate surviving cholinergic PPN neurons. Behavioral testing of transgenic mice showed improvements in postural stability, gait, sensorimotor integration, forelimb akinesia, and general motor activity. In addition, electrophysiological recordings of PPN neurons revealed increased spiking after treatment with DREADDs (Pienaar et al., 2015).

#### 3.1.2 Human studies

While several studies have demonstrated a positive outcome on gait following PPN DBS, findings are equivocal (Bourilhon et al., 2022; Dayal et al., 2021). Potential reasons for the discrepancies observed may derive from the differing time‐point of surgery in the course of the disease and the exact location of electrode placement within the PPN. Thus, because of the lack of a clearly defined target within the PPN, results from these studies must be interpreted with caution. Furthermore, there are differences in how and which (axial) motor symptoms have been assessed. Most studies have used the Unified Parkinson's Disease Rating Scale III (UPDRS III) (Movement Disorder Society Task Force on Rating Scales for Parkinson's Disease, 2003), which is not well suited to measure axial symptoms (Thevathasan et al., 2018). To address this issue, customized questionnaires have been developed to facilitate the comparison of clinical stimulation effects on axial symptoms across studies (Dayal et al., 2021; Ferraye et al., 2010; Thevathasan et al., 2011). Nevertheless, questionnaire‐based assessments often lack intra‐ and inter‐rater reliability. Thus, quantitative measures have been proposed for gait assessment. To this end, postural sway (deviations in center of pressure) has been postulated as a suitable outcome measure. In a small study, involving 13 PD patients with severe clinical balance impairment, PPN stimulation showed an improvement of postural sway in both the medication ON and OFF state (Perera et al., 2018). The long‐term efficacy and safety of unilateral PPN stimulation in PD patients with refractory gait and balance difficulties was assessed in a clinical trial at 2and 4 years post‐surgery using the UPDRS part II. At 2 years, patients reported a significant improvement of FOG compared to baseline. In 4 years, there was no significant change of any item of the UPDRS part II. However, patients reported improvements of falls in both the ON and OFF medication state (Mestre et al., 2016).

A recent meta‐analysis of 13 clinical trials has reviewed the effect of PPN LFS on motor symptoms, FOG, and falls, evaluated with gait specific questionnaires (Yu et al., 2020). The clinical trials included showed substantial heterogeneity with respect to the exact electrode location within the PPN, timespan of clinical follow‐up, patient age, disease duration, stimulation frequency, and daily dosage of LD. No improvement of the classical global motor symptoms (rigidity, tremor, and bradykinesia) was observed. However, virtually all trials reported a significant amelioration of gait and other axial symptoms (Yu et al., 2020). In a double‐blind randomized cross‐over study, nine PD patients with severe gait disorder were assessed 24 h after PPN‐surgery using the UPDRS III, and behavioral gait assessment. Compared with stimulation frequencies of 60–80 Hz, lower frequencies of 10–25 Hz led to an amelioration of akinesia and a reduction of gait difficulties in 7/9 patients (Nosko et al., 2015). The better response to LFS may be partially explained by the electrophysiological properties of cholinergic neurons in the caudal PPN. Along these lines, several patch‐clamp experiments have revealed a plateau phase of cholinergic neurons in the caudal PPN at frequencies of 40–60 Hz, while cholinergic neurons were deactivated at frequencies above 60 Hz (Garcia‐Rill et al., 2015; Simon et al., 2010).

Because of interconnected nuclei, unilateral stimulation likely also exerts effects on the contralateral PPN (Hamani et al., 2016). Unilateral stimulation was also observed to enhance blood flow in the contralateral hemisphere, as evidenced by a PET study involving three patients with advanced PD in the OFF medication state, both at rest and during a lower limb motor task (Ballanger et al., 2009). Conversely, the paired nature of PPN connections suggests that bilateral stimulation may offer greater efficacy in modulating its function (Hamani et al., 2011). Only two studies have directly compared the effects of unilateral versus bilateral PPN DBS (Khan et al., 2012; Thevathasan, Cole, et al., 2012). In a cohort of five PD patients ON medication, Khan et al. reported an improvement of UPDRS motor scores by 5.7% with unilateral stimulation, while the same motor scores were ameliorated by 18.4% with bilateral stimulation (Khan et al., 2012). A second cohort of 17 PD patients was analyzed in a double‐blind study using an objective spatiotemporal gait analysis. DBS induced improvement of gait was twice as good with bilateral compared with unilateral stimulation (Thevathasan, Cole, et al., 2012). That said, findings of these studies should be interpreted cautiously, as the assessment of symptom improvement relied solely on UPDRS III scores (Khan et al., 2012; Thevathasan, Cole, et al., 2012).

Alongside single target stimulation, PPN DBS has also been combined with other targets, including the STN, GPi, and caudal zona inserta. Combining PPN DBS with other targets has the advantage of treating classical motor symptoms and axial symptoms at the same time. Simultaneous bilateral STN DBS and PPN LFS (20–25 Hz) has demonstrated an improvement of UPDRS III scores, falls, postural instability, and FOG in individual PD cases (Ferraye et al., 2010; Plaha & Gill, 2005; Stefani et al., 2007). Similarly, combining PPN DBS with GPi DBS has shown marked effects on gait initiation and FOG in a 66‐year‐old PD patient with severe peak‐dose dyskinesia, ON freezing, and postural instability (Schrader et al., 2013). Notably, both GPi and PPN DBS alone reduced FOG, with PPN DBS being slightly more effective. However, the combination of both targets had a significantly larger impact on FOG compared with single target stimulation (Schrader et al., 2013). Caudal zona inserta stimulation was addressed in a study of seven PD patients with predominant axial symptoms. Bilateral caudal zona inserta and PPN stimulation in combination were superior to single target stimulation in improving both a composite axial subscore and UPDRS III motor scores (Khan et al., 2011). Within the PPN, the caudal part appears to be crucial for the effects of PPN DBS (Thevathasan et al., 2011; Yu et al., 2022), based on the topographical distribution of local field potentials (Tattersall et al., 2014; Thevathasan, Pogosyan, et al., 2012). Additional regional stimulation effects on gait in PD patients have been observed in the cuneiform nuclei and posterior parts of the PPN (pars dissipata and pars compacta), suggesting a slightly better response when stimulating posterior parts of the PPN (Goetz et al., 2019). This finding is in line with pre‐clinical studies, which likewise have reported the best stimulation effects in the posterior part of the PPN (Garciarill, 1991; Gut & Winn, 2015; Reese et al., 1995). Thus, most centers prefer to target the caudal part of the PPN (Hamani et al., 2011). However, given the small size and lack of anatomically defined inner boundaries, most trajectories likely will cover both the caudal and the rostral part of the PPN, enabling selective stimulation.

#### 3.1.3 Summary of PPN DBS

Animal studies clearly suggest a pivotal role of the PPN cholinergic neurons as important drivers of gait control. Furthermore, declining PPN cholinergic tone in the parkinsonian state is linked to deterioration of locomotion and other axial symptoms. PPN DBS ameliorates axial symptoms and at the same time restores PPN cholinergic function, suggesting that DBS effects are relayed mainly through the cholinergic system. This is supported by the effect of AChEIs, such as rivastigmine (Emre et al., 2004), donepezil (Aarsland, 2002), and galantamine (Aarsland et al., 2003), which reduce the frequency of falls in PD patients (Perez‐Lloret et al., 2016).

Clinical data on the effects of PPN DBS in PD patients are still limited. While unilateral stimulation has shown significant improvement of gait, bilateral stimulation appears to be superior, maybe not surprising given the need for bilateral limb activation during locomotion. Even though reports are equivocal, LFS likely is more efficient than HFS, which is in line with data from STN DBS suggesting that lower frequencies are favorable when gait problems are predominant (Conway et al., 2021). PPN DBS is currently performed in PD patients experiencing early and severe FOG, postural instability, gait dysfunction, and may also be an option in patients with LD refractory motor symptoms (Thevathasan et al., 2018). PPN DBS is therefore considered as a rescue‐strategy after the development of severe FOG despite or as a consequence of STN and/or GPi DBS (Schrader et al., 2013). The rate and nature of PPN DBS related risks and side effects appear to be similar to that of conventional DBS. There are no head‐to‐head studies comparing the effects of PPN DBS with STN or GPi stimulation. However, PPN DBS effects on PD cardinal motor symptoms are of a lower magnitude, whereas it is more efficient in the treatment of axial symptoms (Collomb‐Clerc & Welter, 2015). Combining conventional targets such as the GPi or STN with PPN, in our view, is the most viable option in PD patients eligible for classical DBS who show pronounced and LD refractory FOG. That said, stimulation of multiple targets often requires parallel HFS and LFS. Even though some of the currently available DBS devices can handle multiple frequencies to some extent, implantation of two pulse generators may be necessary. Because of its small size and lack of defined anatomical subdivisions, targeting specific cell populations within the PPN is challenging, but stimulation of the caudal portion which inhabits the major portion of cholinergic neurons seems to provide the best clinical outcome. Indeed, there has been extensive research on surgical protocols to define anatomical landmarks to facilitate the precise localization of the PPN and its subregions (Hamani et al., 2011). In this context, the intraoperative use of microelectrode recording, and MRI based visualization techniques have become increasingly important in facilitating electrode placement. However, even though different firing rates of neurons have been recorded during PPN surgery (Shimamoto et al., 2010; Tattersall et al., 2014; Weinberger et al., 2008), it is still challenging to allocate them to specific subregions (Hamani et al., 2011). As the PPN extends along the longitudinal axis of the brainstem, the trajectory will include both the caudal and rostral subregions of the PPN in most cases. That said, intraoperative recordings in combination with test‐stimulation of different contacts are feasible and may lead to the best outcome. PPN DBS effects appear to be sustained over months to years, but data on longtime follow‐up are lacking. A summary of relevant PPN DBS studies can be found in Table 1.

**TABLE 1:** Overview of the optimal stimulation parameters, surgical approach, and patient selection for deep brain stimulation (DBS) of the pedunculopontine nucleus (PPN) and the nucleus basalis of Meynert (NBM) for patients with Parkinson's disease (PD).

Parameter	PPN‐DBS	NBM‐DBS
Patients (studies)	**Nosko et al.:** 9 PD patients (age 64 ± 5.4 years) **Khan et al., 2011:** 7 PD patients (age 40–70 years) **Khan et al., 2012:** 5 PD patients (age 61–65 years) **Thevathasan et al., 2012:** 17 patients (age 64–67 years) **Ferraye et al.:** 6 PD patients (age 47–72 years) **Plaha and Gill et al.:** 2 PD patients (age 60 years) **Stefani et al.:** 6 PD patients (61–69 years) **Schrader et al.:** 1 PD patient (66 years old) **Perera et al.:** 13 PD patients (56–78 years) **Mestre et al.: 9 PD patients** (median age 63 years)	**Bogdan et al.:** 33 PD patients (57–71 years) **Freund et al.:** 1 PDD patient (71 years) **Nombela et al.:** 1 PD patient (68 years) **Cappon et al.:** 5 PDD patients (46–75 years) **Sasikumar et al.:** 6 PD patients (61–70 years) **Gratwicke et al. 2020:** 6 PDD patients, 5 DLB patients (age 65–72 years) **Maltête et al.:** 6 DLB patients (50–69 years)
Motor effects	**Gait, postural instability:** Improvement of postural sway, (Perera et al., 2018), FOG and falls (Mestre et al., 2016), gait difficulties (Nosko et al., 2015)	**No improvement of motor symptoms** (Nombela et al., 2019)
Cognitive effects	**No sufficient data**	**Inconsistent results regarding attention, cognition, concentration:** Improvement of neuropsychological test batteries (Freund et al., 2009; Gratwicke et al., 2018; Nombela et al., 2019), slowing of cognitive decline (Cappon et al., 2022), significant reduction of visual hallucinations (Gratwicke et al., 2018), no improvement in overall cognitive function (Cappon et al., 2022; Gratwicke et al., 2020; Maltête et al., 2021; Sasikumar et al., 2022)
Frequency	**LFS below 10‐25 Hz (superior to HFS above 60 Hz)** (Nosko et al., 2015)	**LFS at 20 Hz preferred** (Nazmuddin et al., 2021); **HFS without negative effects** (Bogdan et al.)
Stimulation paradigm (continuous vs. intermittent)	**No sufficient data**	**Intermittent stimulation (superior to continuous stimulation):** Improvement of sustained attention (Sasikumar et al., 2022)
Hemisphere	**Bilateral stimulation (superior to unilateral stimulation):** Improvement of FOG (Khan et al., 2012; Thevathasan et al., 2012)	**Bilateral stimulation (seems superior to unilateral stimulation, but data is insufficient)** (Gratwicke et al., 2018; Turnbull et al., 1985)
Combination with other targets	**+ STN (no head‐to‐head comparison to single target stimulation):** Improvement of UPDRS III scores, FOG, postural instability (Ferraye et al., 2010; Plaha & Gill, 2005; Stefani et al., 2007) **+ GPi (superior to single target stimulation):** Improvement of gait initiation, FOG (Schrader et al., 2013) **+ CZI (superior to single target stimulation):** Improvement of UPDRS III scores (Khan et al., 2011)	**+ STN (no head‐to‐head comparison to single target stimulation):** Improved attention, concentration, drive, and motor symptoms (Freund et al., 2009) **+ GPi (superior to single target and STN stimulation):** Improved UPDRS III scores, improved performance and neuropsychological tests (Nombela et al., 2019)

Abbreviations: CZI, caudal zona inserta; DBS, deep brain stimulation; FOG, freezing of gait; GPi, globus pallidus internus; HFS, high frequency stimulation; LFS, low frequency stimulation; NBM, nucleus basalis of Meynert; PPN, pedunculopontine nucleus; STN, subthalamic nucleus; UPDRS III, Unified Parkinson's Disease Rating Scale III.

### 3.2 NBM DBS

The utilization of NBM DBS to increase ACh levels in the cortex has been explored as a potential therapeutic approach for improving cognitive symptoms in both PDD and DLB (Baldermann et al., 2018; Gratwicke et al., 2013, 2018). DBS in PD patients with cognitive impairment is of particular significance, as PD patients with major cognitive deficits usually are not eligible for STN DBS because of the risk of cognitive deterioration (Foltynie & Hariz, 2010; Hariz et al., 2000).

#### 3.2.1 Animal studies

Early pre‐clinical studies have demonstrated an increase of cortical ACh mediated by both continuous and intermittent stimulation of the NBM (Casamenti et al., 1986; Kurosawa et al., 1989a, 1989b; Rasmusson et al., 1992). In rats subjected to low frequency NBM stimulation (30 Hz), a 40% rise in ACh release within the parietal cortex was observed (Casamenti et al., 1986). Similarly, continuous LFS (20–50 Hz) in rats led to a twofold increase in cortical ACh (Kurosawa et al., 1989a). Conversely, another study showed a higher release of cortical ACh following HFS (Rasmusson et al., 1992). In contrast to other studies, however, the latter study used pulsed rather than continuous stimulation and added atropine to enhance evoked release of ACh (Rasmusson et al., 1992). A recent meta‐analysis covering four animal studies reported NBM LFS (20–50 Hz) to induce the highest elevation of cortical ACh levels. No differences between continuous and intermittent stimulation were observed with regard to the effects on cortical ACh levels (Nazmuddin et al., 2021). It is believed that a vasodilative effect relays the elevation of cortical ACh levels by increasing cortical blood flow. Along these lines, Biesold et al. have demonstrated that NBM stimulation in anesthetized rats led to an ipsilateral vasodilation of cortical vessels, which could be blocked by nicotinergic and muscarinergic antagonists (Biesold et al., 1989). Beyond the elevation of cholinergic concentration, NBM DBS likely also induces neuronal plasticity. For example, Kilgard and Herzenich have demonstrated that episodic NBM stimulation, paired with concomitant auditory stimuli in adult rats, resulted in a substantial and progressive reorganization of the primary auditory cortex (Kilgard & Merzenich, 1998).

The effects of NBM stimulation on different traits of cognition have previously been summarized in a review including 19 pre‐clinical trials in rodents and non‐human primates (Nazmuddin et al., 2021). The majority of these studies performed their stimulation experiments in wild‐type rats, while two studies used a transgenic AD line in rodents. Cognitive function was measured with different cognitive tasks, including encoding, consolidation, and retrieval. Effects mediated by NBM stimulation were observed mainly on encoding, immediate retention of memory, and speed of learning, while there was no effect on long‐term memory. In general, stimulation‐related effects emerged 24 h after training and lasted for up to 2 weeks (Nazmuddin et al., 2021). The majority of studies (14/19) applied unilateral stimulation (mostly in the right hemisphere), while the remaining studies used bilateral stimulation (Avila & Lin, 2014; Liu et al., 2017, 2018; Mayse et al., 2015). None of these studies directly compared the outcome of unilateral with bilateral stimulation.

Two studies compared a continuous with an intermittent stimulation protocol, reporting a clear superiority of intermittent stimulation (Koulousakis et al., 2020; Liu et al., 2017). Liu et al. compared intermittent and continuous stimulation in adult monkeys, corroborating with this finding. Continuous stimulation was applied as a block of 100 stimulation pulses interleaved with 100 pulses without stimulation. Continuous stimulation worsened working memory performance, while intermittent stimulation led to an improvement (Liu et al., 2017). Overall, the best cognitive improvement of spatial memory performance was observed with intermittent stimulation using biphasic electrical pulses (60/80 Hz) for 20 s interleaved with a pause for 40 s (Nazmuddin et al., 2021). Moreover, two studies in rats and rhesus monkeys, respectively, have revealed positive effects on cognitive performance at frequencies of 60 Hz (Koulousakis et al., 2020; Liu et al., 2018). Conversely, most pre‐clinical studies have used higher frequencies up to 120 Hz to stimulate the NBM and observed stronger cognitive benefits at higher frequencies. Some studies even reported worsening of cognitive performance after reducing the stimulation frequency (Huang et al., 2019; Liu et al., 2017).

#### 3.2.2 Human studies

The first case of NBM DBS reported in PD was a 71‐year‐old PD patient with slowly progressive PDD receiving combined high‐frequency STN stimulation and low‐frequency NBM DBS. High‐frequency STN DBS was administered for 3 months before low‐frequency NBM stimulation was turned on. Isolated STN DBS improved motor symptoms but had no effect on cognitive function. Notably, cognitive performance, including attention, concentration, and drive distinctly improved upon activating NBM stimulation and worsened after stimulation was turned off (Freund et al., 2009). In another report, a 68‐year‐old PD patient diagnosed with mild cognitive impairment underwent DBS surgery targeting the GPi and the NBM using a single electrode per hemisphere. The patient showed an improvement of UPDRS III scores by 61% 2 months after initiation of GPi stimulation. No further motor improvement was observed after NBM stimulation was added. However, combining NBM and GPi stimulation improved performance across various neuropsychological tests. These effects remained stable over 1 year and no side effects were observed (Nombela et al., 2019).

Two randomized cross over clinical trials on NBM DBS in PDD and DLB were reported in 2020 (Gratwicke et al., 2020) and 2021 (Maltête et al., 2021), respectively. Surgery and stimulation were well tolerated. The cognitive assessments did not reveal significant stimulation induced improvements, and even worsening in one study (Maltête et al., 2021). That said, PET and functional MRI provided evidence for a modulation of regions and networks associated with cognitive function (Gratwicke et al., 2020; Maltête et al., 2021). In a randomized double‐blind clinical trial, six patients with PDD and motor fluctuations received either bilateral low‐frequency (20 Hz) NBM stimulation or sham stimulation for 6 weeks, subsequently crossing over to the respective other condition (Gratwicke et al., 2018). The intervention was well tolerated, with no serious adverse events reported. Although no improvements were observed in primary cognitive outcomes, NBM DBS showed an amelioration of scores of the neuropsychiatric inventory compared to sham stimulation. Two patients experienced a significant reduction of visual hallucinations, and three patients reported an improvement of health‐related quality of life (Gratwicke et al., 2018). In a phase‐II double‐blind crossover pilot trial, involving six participants diagnosed with advanced PD and cognitive impairment, Sasikumar et al. investigated the effects of single‐trajectory DBS of GPi and NBM on motor symptoms, cognitive performance, and biomarkers (Sasikumar et al., 2022). As expected, GPi DBS resulted in improvements of dyskinesia and motor fluctuations. NBM DBS in addition to GPi DBS led to reduced metabolism in right frontal and parietal cortical regions and enhanced functional connectivity in volume of tissue activated analysis as assessed by ^18^F‐flourodeoxyglucose PET and magnetoencephalography. However, these findings were not accompanied by concomitant cognitive improvement of PD patients after 1 year (Sasikumar et al., 2022). Another double‐blind cross‐over study including six patients with PDD undergoing NBM LFS (20 Hz) did not report cognitive improvement, but two patients exhibited a stimulation induced slowing of cognitive decline (Cappon et al., 2022).

Most animal and human studies for NBM DBS in PDD and DLB used bilateral continuous low‐frequency stimulation (20 Hz) (Nazmuddin et al., 2021). Although LFS seems to result in a more favorable outcome (Nazmuddin et al., 2021), the proposed negative effects of HFS were challenged by a recent study in 33 PD patients (Bogdan et al., 2022). Patients received GPi stimulation for motor fluctuations and dyskinesia. Because of the anatomical proximity of the GPi to the NBM, the most distal contact was located within the NBM in a subset of patients. Analysis of these patients with an active distal contact and assumed NBM high‐frequency co‐stimulation at 130–185 Hz showed no signs of cognitive decline after 12 months (Bogdan et al., 2022). Given these equivocal findings, suspicion arises that the stimulation paradigm (i.e., intermittent vs. continuous) rather than the frequency applied may be crucial for achieving an optimal stimulation effect. To this end, Sasikumar et al. compared intermittent stimulation consisting of a pulse train (3 mA, 60 μs at 60 Hz), cycling between 20 s ON and 40 s OFF stimulation for 1 h daily, with continuous stimulation using the same parameters. Intermittent stimulation significantly improved sustained attention, whereas continuous stimulation did not affect cognitive scores (Sasikumar et al., 2023). Most pre‐clinical studies have used unilateral NBM stimulation to achieve a cognitive improvement in rodents and non‐human primates. Subsequent clinical trials have either been performed with bilateral stimulation or NBM DBS was combined with other targets. No studies are available, directly comparing the outcome of unilateral versus bilateral NBM DBS.

#### 3.2.3 Summary of NBM DBS

The role of the NBM in cognition has been recognized for decades. The first attempt to modulate cognitive function by electric stimulation was performed by Turnbull et al. in 1984 (Turnbull et al., 1985). A 74‐year‐old patient with AD received unilateral NBM stimulation. After 9 months, ipsilateral cortical glucose levels increased, but cognitive function remained unchanged. Subsequent studies in both DLB and PDD have reported limited improvement of cognitive function and slowing of cognitive decline after NBM DBS, but also worsening of cognition in some cases. NBM DBS in DLB has been shown to modulate brain networks associated with cognition, but accompanying improvement of clinical parameters was mostly lacking. So far, there is no sufficient evidence for a significant and sustained cognitive improvement by NMB DBS in PD. This is particularly true for available randomized controlled trials. Even though the NBM is a key factor in cognitive function, the complex and widespread brain changes associated with altered cognition in PD (Mihaescu et al., 2019) may explain the somewhat equivocal findings obtained from NBM DBS studies. Regarding complication rates and side effects of NBM DBS, the limited data available do not suggest an increased risk compared with conventional DBS. Moreover, NBM DBS is rarely performed in a single target approach. Thus, it is difficult to provide an estimate of the target related risk.

Patients included in the available trials generally showed mild‐to‐moderate cognitive impairment. It is unclear if NBM DBS also is effective in more severely cognitively impaired PD patients. Clinical trials in AD patients suggest that NBM DBS may be efficacious in patients with severe cognitive impairment (Picton et al., 2024), but long‐term data are scarce and inconclusive, and studies in severely affected PDD/DLB individuals are lacking. In the light of the limited evidence of NBM DBS efficacy in PD and largely lacking amelioration of PD motor symptoms, single target NBM DBS, in our view, is not recommended and should be applied in combination with traditional targets, preferably in the framework of clinical studies. Currently, a randomized, sham‐controlled trial is investigating combined STN and NBM DBS for PDD (DEMPARK DBS), with safety as the primary outcome and effects on cognition, daily functioning, motor skills, mood, caregiver burden, and economic aspects as a secondary outcome (Daniels et al., 2020). It is unclear whether NBM DBS should preferably be used in combination with either GPi or STN DBS. Both, GPi and STN DBS have been shown to ameliorate motor symptoms with STN DBS being superior in reducing dopaminergic medication after surgery (Bronstein et al., 2011; Follett et al., 2010). STN DBS led to a faster decline of Mattis Dementia Rating Scale scores (Weaver et al., 2012). Conversely, there were two other trials not observing a different cognitive outcome between STN and GPi DBS after 12 months and 3 years, respectively (Boel et al., 2016; Odekerken et al., 2015). With regard to the cognitive outcome and other non‐motor symptoms such as depression, combining NBM DBS with the GPi is preferred based on experts' consensus and the largest randomized study (Bronstein et al., 2011; Follett et al., 2010).

With respect to the identification of optimal stimulation parameters, there are conflicting results from animal and human studies, respectively. Whereas most of both rodent and non‐human primate studies suggested greater efficacy with HFS, the first studies in human PD suggested that stimulation at 20 Hz may be beneficial. However, this has been challenged recently, and no clear recommendation can be made at this point. DBS efficacy appears to be better if intermittent rather than continuous stimulation paradigms are applied. That said, even though closed loop stimulation paradigms are on the rise for conventional DBS (Stanslaski et al., 2024), adaptive stimulation is not feasible at this point. While most studies applied unilateral stimulation, bilateral stimulation has been favored by more recent study designs. Lastly, the majority of pre‐clinical NBM DBS studies was performed in either wild‐type animals or AD models. Thus, the transferability to PD/DLB remains unclear. We have summarized the findings of the major NBM DBS studies in Table 1.

## 4 CONCLUSION

A‐syn mediated neurodegeneration of the PPN and NBM is key in the pathogenesis of cholinergic deficiency in PD. Pre‐clinical and clinical studies support a strong association of cholinergic degeneration with both gait impairment and cognitive deficits. Notably, the foundation of gait impairment appears to lie in the deterioration of the attentional cognitive domain rather than motor system deficiency. Furthermore, there are versatile interactions between the dopaminergic and cholinergic systems, which should be accounted for when aiming to target specific motor and non‐motor symptoms in PD.

PPN DBS has been proven to modulate the cholinergic tone and is associated with improvement of axial symptoms, most importantly FOG unresponsive to dopaminergic treatment. Conversely, NBM DBS has shown its merit in partially improving cognition in cognitively impaired PD patients, albeit evidence from human studies is still limited and equivocal. Given the circumscribed effects of both PPN and NBM DBS, in our view, both targets should be combined with either STN or GPi DBS. PPN and NBM DBS demand specific stimulation paradigms, whereby data on the recommended settings to achieve optimal clinical outcome are still conflicting in many aspects. Neither PPN nor NBM DBS can be regarded as standard clinical care at his point and thus should be performed by experienced centers, preferably in the context of clinical trials, to enable recommendations for a potentially broader clinical application. Ideally, future studies should directly compare unilateral versus bilateral stimulation and pay particular attention to stimulation frequencies, for example, in cross‐over trials. Lastly, including recent advances in stimulation techniques such as mono‐segmental stimulation (“steering”) and in vivo assessment of local field potentials (“sensing”) in clinical trials may shed further light on the optimal stimulation site and improve clinical outcome.

## AUTHOR CONTRIBUTIONS

**V. Witzig:** Conceptualization; writing – original draft; funding acquisition; project administration; visualization; writing – review and editing; investigation; methodology; validation; data curation. **R. Pjontek:** Writing – review and editing; writing – original draft; visualization; investigation; methodology. **S. K. H. Tan:** Conceptualization; writing – review and editing. **J. B. Schulz:** Writing – review and editing; supervision; resources. **F. Holtbernd:** Conceptualization; supervision; writing – review and editing; project administration; validation.

## CONFLICT OF INTEREST STATEMENT

JBS is working in the advisory board of Forward Pharma, MSD, Lundbeck, Biogen, Eisai, Novo Nordisk, Roche, Reata, and Lilly. JBS has received reimbursement for lectures from Merz, Teva, Bayer, UCB, Lilly, Boehringer, GSK, Bial, Novartis, Biogen, and Eisai. JBS has received grants for research projects from Biogen, Eisai, and Lilly. FH received travel and conference fees from Bial, Desitin, Abbott, Zambon, and Abbvie. VW received travel and conference fees from Medtronic. The other authors have no conflicts of interest to declare.

### PEER REVIEW

The peer review history for this article is available at https://www.webofscience.com/api/gateway/wos/peer‐review/10.1111/jnc.16264.

## Data Availability

Data sharing is not applicable to this article because no new data were created or analyzed in this study.
